# Template-free generation and integration of functional 1D magnetic nanostructures†

**DOI:** 10.1039/d3nr03878e

**Published:** 2023-11-09

**Authors:** Mehran Sedrpooshan, Claudiu Bulbucan, Pau Ternero, Pierfrancesco Maltoni, Calle Preger, Simone Finizio, Benjamin Watts, Davide Peddis, Adam M. Burke, Maria E. Messing, Rasmus Westerström

**Affiliations:** a NanoLund, Lund University Box 118 221 00 Lund Sweden rasmus.westerstrom@sljus.lu.se; b Synchrotron Radiation Research, Lund University Box 118 221 00 Lund Sweden; c MAX IV Laboratory, Lund University Lund SE-22100 Sweden; d Solid State Physics, Lund University Box 118 221 00 Lund Sweden; e Department of Materials Science and Engineering, Uppsala University Box 35 751 03 Uppsala Sweden; f Ergonomics and Aerosol Technology, Lund University Lund SE-22100 Sweden; g Paul Scherrer Institut 5232 Villigen PSI Switzerland; h Institute of Structure of Matter, National Research Council (CNR) Monterotondo Scalo 00015 Rome Italy; i Department of Chemistry and Industrial Chemistry, University of Genova 16146 Genova Italy

## Abstract

The direct integration of 1D magnetic nanostructures into electronic circuits is crucial for realizing their great potential as components in magnetic storage, logical devices, and spintronic applications. Here, we present a novel template-free technique for producing magnetic nanochains and nanowires using directed self-assembly of gas-phase-generated metallic nanoparticles. The 1D nanostructures can be self-assembled along most substrate surfaces and can be freely suspended over micrometer distances, allowing for direct incorporation into different device architectures. The latter is demonstrated by a one-step integration of nanochains onto a pre-patterned Si chip and the fabrication of devices exhibiting magnetoresistance. Moreover, fusing the nanochains into nanowires by post-annealing significantly enhances the magnetic properties, with a 35% increase in the coercivity. Using magnetometry, X-ray microscopy, and micromagnetic simulations, we demonstrate how variations in the orientation of the magnetocrystalline anisotropy and the presence of larger multi-domain particles along the nanochains play a key role in the domain formation and magnetization reversal. Furthermore, it is shown that the increased coercivity in the nanowires can be attributed to the formation of a uniform magnetocrystalline anisotropy along the wires and the onset of exchange interactions.

## Abstract 2

This work reports on a novel template-free technique for producing isolated 1D magnetic nanochains and nanowires directly onto substrates and device architectures by directed self-assembly of gas-phase-generated nanoparticles.

## Graphical abstract

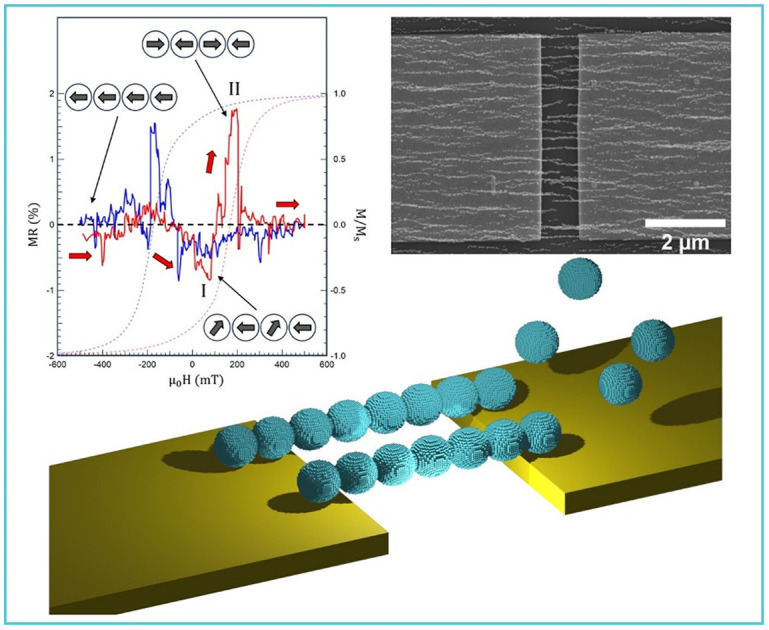

## 1. Introduction

Cylindrical 1D magnetic nanowires (NWs) exhibit large axial shape anisotropy due to the high aspect ratio and can host novel magnetization configurations resulting from the curved geometry and absence of lateral confinement.^1,2^ Owing to their unique properties, magnetic NWs have attracted significant interest, not only because of the promise of new physical phenomena but also due to their great potential as components in magnetic storage and logic devices,^3–5^ spintronic applications,^6–9^ and novel permanent magnets.^10,11^ Magnetic NWs are commonly generated using template-assisted electrochemical deposition, which offers great versatility in terms of size, geometry, and composition^12–14^ but relies on templates that have to be dissolved and require additional transfer steps to deposit the NWs onto substrates for characterization or integration into devices. Direct-write fabrication of 3D magnetic nanostructures,^15,16^ including NWs,^17–19^ can be achieved by focused electron beam-induced deposition (FEBID). However, while providing large flexibility with respect to available geometries that can be produced, the technique typically suffers from impurities and poor crystallinity.^15,17^

Linear 1D arrangement of self-assembled nanoparticles (NPs) has structural similarities to NWs in that they possess a large aspect ratio and curved geometry. These so-called nanochains (NCs) have applications in bio-medicine,^20,21^ as contrast agents,^22^ microwave absorption,^23,24^ catalysis,^25,26^ plasmonics,^27,28^ memory applications,^29^ and magnetic field and gas sensing.^30,31^ Self-assembly of functional magnetic NPs onto electric circuits offers a scalable and low-cost approach for producing novel devices.^32–36^ However, studies have mainly focused on 2D particle films requiring multi-stage device fabrication that can suffer from low uniformity and cracks of the particle layer, which are the major drawbacks for large-scale applications.^37–39^ Most self-assembly techniques are based on NPs suspended in a solution where they organize into 1D structures *via* interparticle interactions mediated by attached ligand molecules, templates, or external fields.^40,41^ The latter approach is commonly used to form NCs of magnetic NPs by applying a magnetic field directly to the suspension or during drop-casting onto a substrate.^42–49^ This strategy is simple, cheap, and scalable but generally offers poor control of the spatial distribution of the 1D structures when transferred to a substrate where chain aggregation leads to densely packed assemblies along the surface as the solvent evaporates. An alternative route is to use magnetic fields to form NCs from gas-phase-generated NPs.^25,26,50,51^ Similar to most solution-based methods, these approaches typically result in large bundles of NCs with limited control of the final geometry. Future applications will require precise control of the self-assembly and, ideally, direct integration of the NCs into devices. However, positioning NPs into separated NCs along pre-defined directions onto substrates has proven to be a great challenge,^40^ and recent progress relies on functionalizing the NPs to avoid aggregation,^42^ top-down lithography to spatially confine the suspension,^27,30,52,53^ or 3D nanoprinting combining charged aerosol NPs and electric fields.^54,55^

We have developed a template-free technique for directed self-assembly of magnetic NPs into parallel NCs with controllable average length directly onto most substrates.^56^ The method employs an aerosol technique based on spark ablation^56^ to produce charged NPs suspended in a carrier gas. The NPs are attracted to a substrate using an electric field, where they are self-assembled, one by one, into 1D NCs along the direction of an applied magnetic field. Since the magnetic field is applied independently of the electric field capturing the NPs, the self-assembly can be controlled by varying the direction of the applied magnetic field to generate NCs oriented vertically or along the substrate surface. Furthermore, by varying the external magnetic field direction during the particle deposition, complex vertical and in-plane structures made up of interconnected perpendicular NCs can be generated.^56^ The approach is highly versatile as it relies on charged aerosolized NPs, which various methods can produce including, using precursors, atomizing particles from a chemical solution, flame spray synthesis, or evaporation methods.^57^ In particular, the generation techniques based on spark ablation, used in this work, offer a large selection of building blocks to self-assemble, ranging from single-element^56^ and alloyed^58,59^ NPs to bimagnetic systems.^60,61^

Here, we demonstrate how the approach can be extended to template-free generation of magnetic Co NWs directly along surfaces by post-annealing the NCs. With increasing annealing temperature, the NPs start to fuse and gradually transform into diameter-modulated NWs and, finally, continuous cylindrical-like wires. The method is not limited to assembling NPs along solid surfaces but also provides template-free generation of free-standing 1D nanostructures. Moreover, the technique allows for the direct integration of NCs and NWs onto substrates relevant to applications and different device architectures. The latter is demonstrated by self-assembling Co NCs directly onto a pre-patterned Si chip to produce devices exhibiting magnetoresistance (MR). Similar to 2D particle films,^34,62–64^ the observed MR is attributed to the nanogranular structures of the NCs, where particles along the chains can adopt different magnetization configurations depending on the strength and directions of the applied field, resulting in changes in the conductance due to spin-dependent scattering. To the best of our knowledge, this is the first time NCs have been used as sensing elements in devices exhibiting magnetoresistance (MR). These results demonstrate the potential for future facile on-chip integration of 1D nano-granular MR sensors with integrated circuits.

Furthermore, the generation technique allows for studying the effect on the magnetic properties as the NPs are arranged into parallel NCs and fused into NWs. Previous studies have revealed enhanced magnetic properties as the NPs are arranged into NCs.^42,44,46^ However, these studies have focused on Fe–oxide systems where the magnetization reversal is mainly determined by the shape anisotropy resulting from the high aspect ratio of the NCs. We show that arranging the NPs into parallel 1D linear assemblies leads to highly anisotropic systems with an almost 100% increase in the remanent magnetization compared to 3D networks of randomly oriented and interconnected chains. Moreover, fusing the NCs into NWs results in a 35% increase in the coercivity which cannot be explained by shape anisotropy alone. Combining magnetometry, X-ray microscopy, and micromagnetic simulations, we show that the magnetocrystalline anisotropy (MCA) of the individual NPs and the local geometry plays a decisive role in the magnetization reversal and the formation of magnetic domains.

## 2. Experimental and Simulations

Nanoparticle generation started from the evaporation of materials by repetitive sparks (≈80 Hz) between two high-purity metallic Co rods under the flow of carrier gas.^65^ In this work, a carrier gas composed of 95% N_2_ and 5% H_2_ (1.68 lpm) was used to avoid oxidation, and the pressure in the system was set to 1015 mbar and kept constant during the generation process.^59^ The evaporated material condenses into sub-10 nm primary particles that collide forming larger irregular agglomerates which are then passed through a Ni^63^ bipolar diffusion charger to obtain an even charge distribution, needed for the size selection of the produced NPs. The agglomerates are then transported through a tube furnace where they are compacted into faceted single-crystalline NPs at ≈1473 K before being size-selected to electrical mobility diameters of 40 nm using a differential mobility analyzer. In this work, the particle concentration was monitored online with a TSI Electrometer Model 3068*B*, and the desired coverage was calculated from here.^66^

**Fig. 1:**
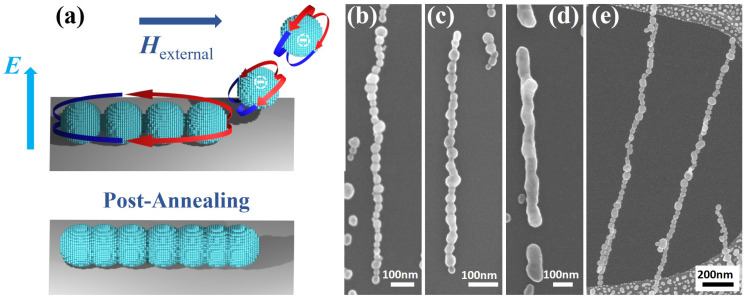
(a) Schematic illustration of NC formation by attracting negatively charged particles using an electric field 
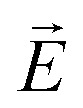
 from the gas phase and self-assembling them by employing an external magnetic field 
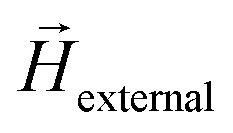
. (b) SEM images of an as-prepared NC, and post-annealed NWs at (c) 673 and (d) 773 K. (e) Free-standing NCs deposited on a TEM grid.

The deposition takes place in an electrostatic precipitator,^67^ where a 750 kV m^−1^ electric field attracts the charged NPs to the substrate. The directed self-assembly occurs when an external magnetic field is applied during the deposition. Directed self-assembly occurs as an external magnetic field magnetizes the NPs along a specific direction, leading to attractive anisotropic magnetic dipole–dipole interactions between the already deposited NPs and the NPs arriving from the carrier gas and the formation of NCs along the field direction, as described in detail here,^56^ and schematically illustrated in Fig. 1(a). In the present study, 1D NCs were generated by applying a magnetic field of 300 mT along the substrate surface. The post-annealing of NCs was carried out at 773 K for 2 min with the same gas composition as the carrier gas.

Crystal structure of the as-prepared and annealed particles was evaluated by X-ray diffraction in transmission geometry using Stoe Stadi MP, Mythen 1k detector, Cu–K alpha radiation, *λ* = 1.54178 Å. Electron microscopy was performed using transmission electron microscopy (TEM) Jeol JEM-3000F, and scanning electron microscopy (SEM), Hitachi-SU8010 Cold Field Emission. To study the magnetic behavior of the structures, they were prepared directly on single crystalline SiO_2_ stripes and the measurements were done using a superconducting quantum interference device vibrating sample magnetometer (SQUID-VSM, MPMS 3, Quantum Design), and a commercial vector vibrating sample magnetometer (MicroSense Model 10 VSM) equipped with a rotating electromagnet generating a maximum field of 2 T. The scanning transmission X-ray microscopy measurements were performed at the PolLux beamline^68^ of the Swiss Light Source and the SoftiMAX beamline^69,70^ of the MAX IV laboratory.

Micromagnetic simulations were conducted by Ubermag with OOMMF micromagnetic calculator.^71,72^ The designed structures were discretized into cubic cells with a 2 nm side length well below the exchange length of face-centered cubic (fcc) Co (≈3.25 nm). An exchange constant of 1.3 × 10^−11^ J m^−1^, cubic anisotropy of 2.7 × 10^5^ J m^−3^, and saturation magnetization of 1.4 × 10^6^ A m^−1^ were used corresponding to Co-fcc phase. The cubic anisotropy was introduced in the nanochain system in random orientation for each particle and also in a uniform manner with an easy axis along the chain. In the nanowire system, this anisotropy was considered with the same orientation throughout the structure with an easy axis along the wire axis.

Magnetoresistive devices are fabricated by the magnetic-field directed self-assembly of ≈40 nm Co NPs on metallic leads with 1 μm and 500 nm gap distances. The leads with 5 μm width are fabricated on SiO_2_/Si wafers *via* electron beam lithography (EBL, Raith-150), and evaporation of a 15 nm Ti adhesion layer followed by 85 nm Au using a Temescal e-beam evaporator. Producing seven devices on the same Si chip required about 90 min of EBL processing and 20 min for self-assembling nanoparticles across the gaps separating the source and drain. Subsequently, the measurements are carried out in a dilution refrigerator equipped with a superconducting magnet. Yokogawa GS200 is utilized as DC voltage source and the current is measured through a Femto DLPCA-200 I/V converter at a 1 nA V^−1^ gain connected to an HP 34401A multimeter.

## 3. Results and discussion

Directed self-assembly of aerosolized magnetic NPs suspended in a carrier gas is achieved by combining electric and magnetic fields. The role of the electric field is to attract the charged particles to the substrate, whereas the magnetic field magnetizes the NPs along a specific direction. As the NPs approach the surface, they are attracted to the already deposited NPs *via* magnetic dipole–dipole interactions to form NCs along the magnetization direction. It should be stressed that the electric field is solely responsible for collecting the aerosolized NPs onto the surface. Consequently, the magnetic field can be applied independently to guide the self-assembly vertically or along any horizontal direction.^56^ Note that the self-assembly is not limited to one direction, but also more complicated 3D vertical structures and tailored 2D interconnected networks can be generated by varying the external magnetic field direction during the deposition.^56^

Herein, we will focus on metallic Co NCs self-assembled along the substrate surface and transformed into NWs by post-annealing. The NCs are generated from Co NPs with an average diameter of 40 nm deposited onto a Si wafer while applying an in-plane magnetic field. The process is schematically illustrated in Fig. 1(a), together with SEM images showing as-produced NCs and NWs post-annealed at different temperatures (Fig. 1(b)–(d)). After annealing the NCs at 673 K for 2 min, the NPs start to fuse at the interparticle boundaries to form what can be described as strongly diameter-modulated NWs. The NPs continue to merge with increasing temperature, resulting in a more uniform cylindrical-like NW at 773 K. The length of the NWs is approximately determined by that of the NCs, which in turn is governed by the particle coverage. However, there is a large tendency to form chain bundles with increasing coverage, and the length of single-particle-wide NCs is limited to about 1 μm. Furthermore, the technique can generate free-standing 1D structures, as can be seen in Fig. 1(e), where NCs self-assembled along the surface of a TEM grid are transversing holes in the carbon film. Since the NCs are freely suspended, NPs extracted from the carrier gas will either pass straight through or contribute to the self-assembly, allowing for significantly more particles to be deposited onto the substrate without forming bundles and consequently the generation of longer chains that can extend several microns.

### 3.1. Structural characterization

**Fig. 2:**
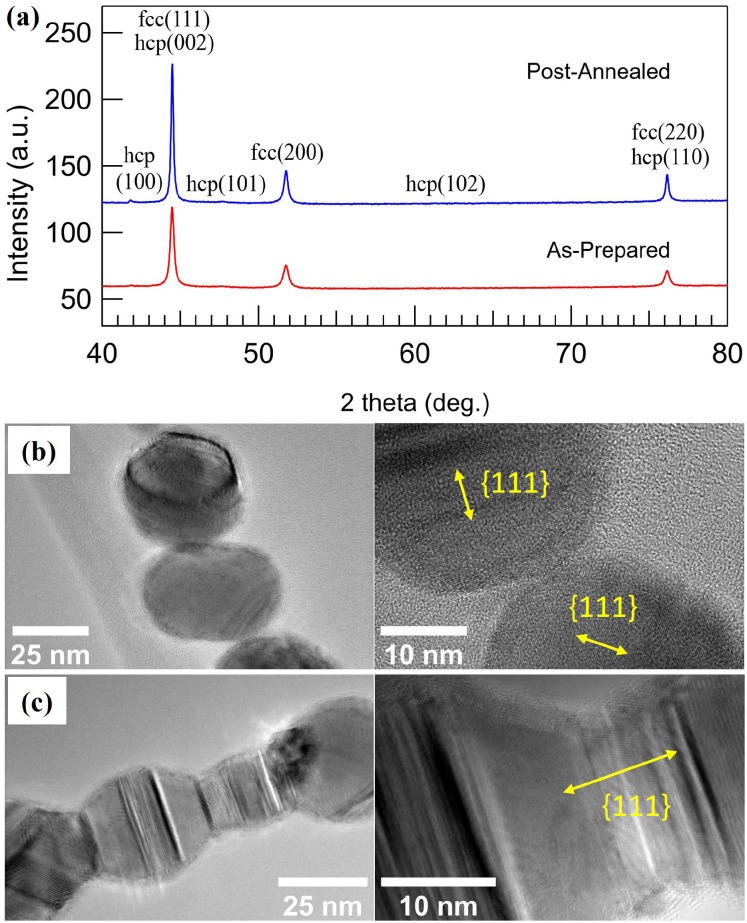
(a) XRD diffractograms of as-prepared, and post-annealed Co nanoparticles, HRTEM images of (b) NCs, and (c) NWs formed by post-anenaling at 773 K for 2 min.

The crystal structure of the nanoscale building blocks making up the 1D structures was determined from XRD measurements performed on a Co NP sample prepared without size selection to maximize the particle concentration and thus diffracted intensity. The diffractogram in Fig. 2(a) shows predominately Bragg peaks at 2*θ* positions corresponding to the metastable Co-fcc phase, with only weak intensity observed from hexagonal close-packed (hcp) Co at low angles. The stabilization of the Co-fcc phase can be understood from the high heating and cooling rates experienced by the particles as they are generated through a liquid-like coalescence of very fine particles at high temperatures and fast cooling to room temperature.^73–75^ The positions of the Bragg peaks do not change as the sample is heated to 773 K for 2 min, indicating that post-annealing does not affect the crystal structure. However, while the symmetry of the crystal structure appears to be unchanged, HRTEM measurements indicate that the interparticle crystallographic alignment along the NCs is affected as the NPs are merged into NWs. Fig. 2(b) displays an HRTEM image from a NC made up of NPs appearing single-crystalline with facets and average 0.208 Å interplanar distances corresponding to {111} (for more images and fast Fourier transform diffractogram see Fig. S1†). Moreover, it appears that there is no interparticle crystalographic alignment along the chain. In contrast, the HRTEM image from a NW (post-annealed NC at 773 K for 2 min), Fig. 2(c), reveals that the NPs have fused at the interparticle junction with a uniform crystal structure. These findings indicate that the post-annealing aligns the crystal lattices along the NCs, consistent with previous studies where polycrystalline Co NW generated by FEBID became single-crystalline after post-annealing at similar temperatures.^18^

### 3.2. Magnetic properties

**Fig. 3:**
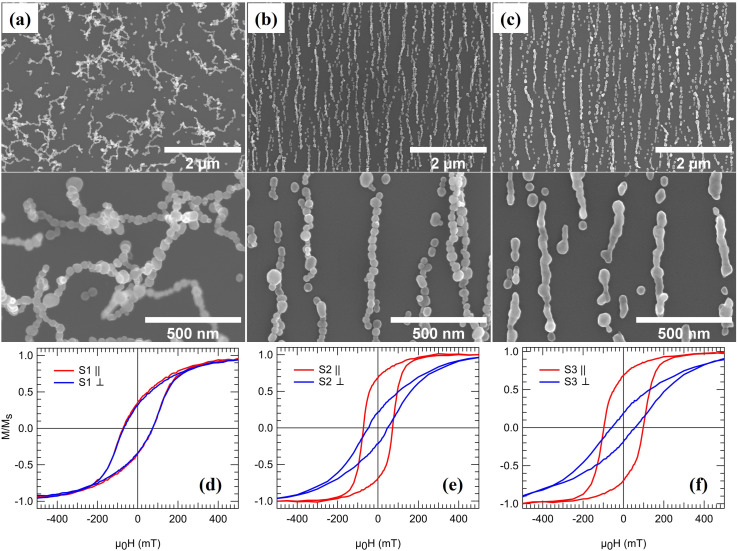
SEM images of (a) randomly oriented interconnected chains, S1, (b) ordered NCs, S2, and (c) continuous NWs, S3, on quartz substrates. Magnetic hysteresis loops of (d) S1, (e) S2, and (f) S3 measured with an in-plane magnetic field oriented parallel (∥) and perpendicular (⊥) to the longitudinal direction of the NCs and NWs.

The generation technique offers a unique possibility of studying the effect on the magnetic properties as the NPs are arranged into linear 1D NCs and subsequently fused to form NWs. Fig. 3(a)–(c) shows three samples, from hereon referred to as S1, S2, and S3, composed of 3D networks of randomly oriented interconnected single chains and bundles (S1), parallel NCs generated *via* magnetic-field assisted self-assembly (S2), and NWs formed by post-annealing (S3). The particle coverage is the same for all three samples and was chosen to obtain a high density of 1D structures to maximize the signal for the magnetometry measurements while avoiding significant bundle formation. The NCs and NWs have large variations in lengths and co-exist with much smaller structures such as dimers, trimers, and single NPs. However, as will be demonstrated, the magnetization reversal becomes independent of length for relatively short NCs and NWs. Consequently, differences between the samples can be directly correlated with aligning the NPs into parallel NCs and forming NWs by post-annealing. Fig. 3(d)–(f) displays magnetization curves recorded from the three samples while applying a magnetic field along one of two orthogonal in-plane directions. Whereas the total magnetic moment at 1.8 T (*m*_1.8 T_) is almost identical for the three samples (Table 1), the remanence and coercivity are significantly different.

**Table 1:** Experimental values of the three samples. The values are obtained at 300 K unless specified otherwise

Sample	*μ*_0_ *H*_c_∥ (mT)	*μ*_0_ *H*_c_⊥ (mT)	*M*_r_/*M*_s_∥	*M*_r_/*M*_s_⊥	*m*_1.8T_ (nA m^2^)	*H*_c_∥ (2 K) (mT)
S1	73.6	71.9	0.35	0.33	135	155.2
S2	72.2	47.2	0.69	0.22	125	170.5
S3	97.3	58.0	0.69	0.19	137	279.0

For S1, the NPs arrange in randomly oriented 3D interconnected chains and bundles. The average particle size is below the single-domain critical diameter for Co-fcc (*D*_c_ ≈ 80 nm), and the NPs can be spontaneously magnetized in the carrier gas as they are cooled down to room temperature. Consequently, they will be attracted to the already deposited NPs *via* magnetic dipole–dipole interactions when reaching the substrate. Since the particle magnetization has no preferential direction, the NPs self-assemble into 3D irregular structures^56^ as opposed to the ordered 1D NCs obtained when applying a magnetic field (S2). The random distribution of chain-like structures leads to an isotropic magnetic response, as demonstrated by the identical shapes of hysteresis loops recorded along two orthogonal in-plane directions, see Fig. 3(d). The high aspect ratio of the NCs in S1 leads to significant shape anisotropy with a magnetic easy-axis parallel to the long axis of the chains. In contrast, the cubic magnetocrystalline anisotropy of the individual Co-fcc NPs results in easy axes along the 〈111〉 directions, which according to HRTEM data in Fig. 2(b), can be randomly oriented along the chains. Consequently, applying an external field to the randomly oriented NCs and bundles in S1 leads to competition between the magnetocrystalline and shape anisotropy. The sample magnetization saturates for sufficiently large external fields as the individual particle moments align along the applied field direction. However, the shape anisotropy dominates at low external fields and rearranges the magnetization along the randomly oriented chains. The net remanent magnetization is thus significantly reduced with a value of *M*_r_/*M*_s_ ≈ 0.34 at 300 K. The observed value is lower than that of *M*_r_/*M*_s_ = 0.50 expected for an isotropic distribution of the magnetic easy axes defined here by the longitudinal direction of the chains, indicating that additional effects influence the remanent magnetization, such as local fields from the randomly oriented neighboring structures.

**Fig. 4:**
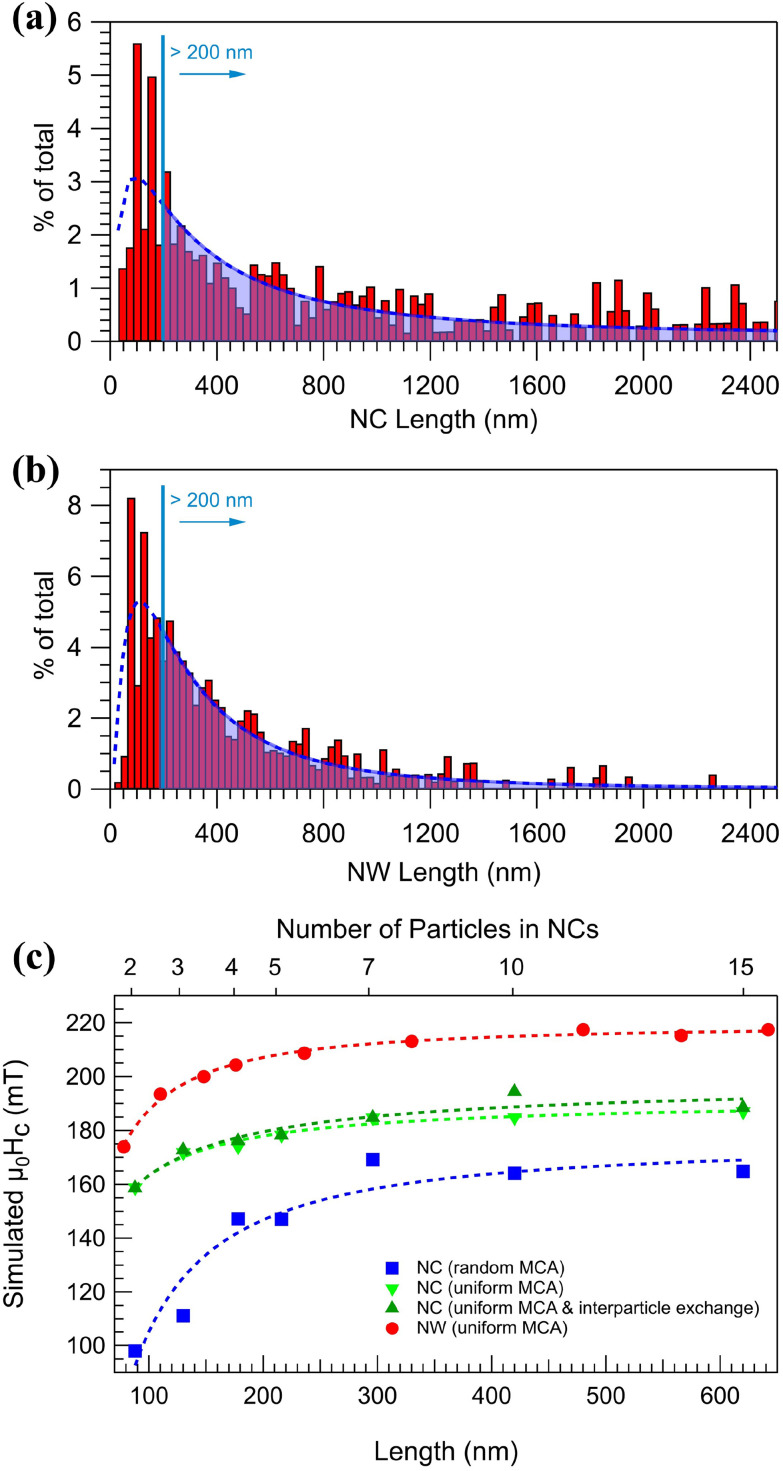
Histograms displaying the percentage of the total number of 1D structures (weighted by area) exhibiting a specific length for NCs (a) and NWs (b). The bottom axis shows calculated length assuming an average 40 nm diameter, the curves are guide to the eye. (c) Simulated coercivity values of NCs and NWs per length.

In contrast, arranging the NPs into parallel NCs (S2) results in a highly anisotropic system, as evident from the significantly different coercive fields *H*_c_ and reduced magnetization *M*_r_/*M*_s_ along and perpendicular to the chain-axis, see Fig. 3(d) and (e) and Table 1. Measuring with the applied field along the chains results in an almost 100% increase in the remanent magnetization compared to S1. The increase can be understood again by the effect of the shape anisotropy, which is colinear with the NCs and the applied magnetic field. On the other hand, fusing the NPs to form NWs results in a ≈35% increase in the coercivity compared to S2. The increase in coercivity can not only be attributed to the shape anisotropy, as it scales with aspect ratio. Fig. 4(a) and (b) shows the size distribution of structures in S2 and S3 obtained by SEM image analysis over a large representing area (see the ESI†) and plotting the relative covered area by NCs or NWs with different sizes. It is observed that in the average size of the structures, the peak position in Fig. 4(a) and (b), does not vary considerably after the post-annealing to be accounted for the changes in coercivity and thus effects other than shape anisotropy must be responsible for the increased coercivity that is investigated by micromagnetic simulations. There is, however, a small decrease in the number 1D structures with length >1200 nm in Fig. 4(b) which can be attributed to the fragmentation of NWs during the post-annealing.^76,77^

#### 3.2.1. Micromagnetic simulations

The differences in coercivity between S2 and S3 were studied by micromagnetic simulations using Ubermag with OOMMF micromagnetic calculator.^71,72^ The NCs were generated by forming a linear 1D arrangement with the particles in direct contact, whereas neighboring NPs were merged by introducing a 10 nm overlap at the interparticle boundaries to mimic the NW structures in S3. Different lengths were obtained by adding NPs with diameters randomly selected in the range of 30–50 nm to account for the size distribution observed in the samples. As indicated by the HRTEM images in Fig. 2, while there is a small separation between particles in the NCs, in NWs particles are fused together at the junctions. Moreover, no significant interparticle crystalline alignment along the NCs is expected as opposed to the NWs, where the post-annealing appears to produce single-crystalline 1D structures. Based on these observations, two main differences between S2 and S3 can be identified that can affect the magnetic properties. Firstly, forming continuous single-crystalline NWs introduces exchange interactions throughout the structure. Secondly, the single-crystalline NW structure is predicted to have mostly uniform MCA as opposed to the NCs, where a predominantly random cubic anisotropy is expected. Fig. 4(c) displays the coercivity values obtained by simulating hysteresis loops parallel to the long axis of NCs and NWs with different lengths. In these simulations, exchange, cubic anisotropy, and demagnetization energies were considered in addition to the Zeeman energy of a sweeping magnetic field. For NCs with random cubic anisotropy, the coercivity increases as more NPs are added up to a length of ≈200 nm, after which it stays constant at around *μ*_0_*H*_c_ = 160 mT, in good agreement with the experimental value of *μ*_0_*H*_c_ = 170 mT measured at 2 K from S2, shown in Table 1. Fusing the NPs to form NWs with uniform cubic anisotropy and longitudinal easy axis results in a similar trend, but now with a ≈30% increase in the coercivity with a constant value of *μ*_0_*H*_c_ = 217 mT. The simulated value is similar to previous computational studies of cylindrical Co-fcc NWs with similar diameters,^78^ and in reasonable agreement with the measured value of 279 mT from S3 at 2 K. The SEM image analysis on images shown in Fig. S3 of the ESI† are presented in Fig. 4(a) and (b) revealing that ≈79% of the NCs and ≈71% of the NWs have lengths corresponding to the constant *μ*_0_*H*_c_ values in Fig. 4(c) indicating that the wide length distribution of the two samples does not significantly influence the magnetic properties. In addition, coercivity values of NCs with uniform MCA with and without interparticle exchange interaction are displayed in Fig. 4(c) that are placed between the other two aforementioned curves. Therefore, the micromagnetic results suggest that the increase in the coercivity of NWs with regard to NCs is achieved by the synergy of exchange interaction enhancement and uniform MCA induced by post-annealing. Furthermore, simulations are consistent with an alignment of the particle crystal lattice and the formation of single-crystalline NWs by post-annealing.

#### 3.2.2. Scanning transmission X-ray microscopy

**Fig. 5:**
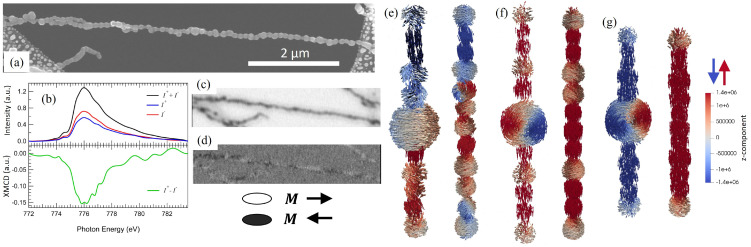
(a) SEM image of a freestanding NC, (b) nano-XAS and XMCD recorded from the NCs. (c) STXM, and (d) STXM-XMCD images of the NC. Simulated equilibrium magnetization configuration after perpendicularly magnetizing NCs with and without the inclusion of a multi-domain particle, with (e) random cubic anisotropy, and (f) uniform cubic anisotropy. (g) The simulated equilibrium state of a NC with fused particles and uniform cubic anisotropy. The red–blue color code represents the projection of the magnetization along the *z*-direction parallel to the chain axis.

Domain formation and magnetization reversal along individual NCs were studied using STXM combined with X-ray Magnetic Circular Dichroism (XMCD). The STXM images were performed by raster scanning the sample across a nano-focused X-ray beam while detecting the transmitted intensity. Before the X-ray measurements, the sample was pre-characterized using SEM. Fig. 5(b) shows a nano-XAS from NCs, recorded at the Co *L*_3_-edge using right and left circularly polarized X-rays. The difference in absorption between the two helicities provides an XMCD spectrum proportional to the projected 3d magnetization onto the direction of the X-ray beam. Mounting the sample at a 30-degree angle to the X-ray beam and tuning the photon energy to the maximum *L*_3_ XMCD signal thus provides spatially resolved XMCD images with a black and white contrast corresponding to the magnetization direction along the 1D structures.

Fig. 5(a) and (c) display an SEM and an STXM image from a free-standing NC self-assembled directly along a TEM grid and post-annealed at 776 K for 2 min. While fusing has started at some of the interparticle boundaries, most NPs still maintain their original shape, and the structure can be expected to be more NC than NW-like. Before the measurement, the sample was magnetized perpendicular to the chain axis using a 7 T *ex situ* field. The remanent state, shown in Fig. 5(d), was imaged corresponding to the equilibrium magnetic configuration adopted by the system as it relaxes after the field have been removed. The appearance of bright and dark XMCD contrast demonstrates the presence of domains with the magnetization predominately lying along the chain axis but in different directions. Considering that the NC is far from uniform, it is likely that the emergence of magnetic domains originates from local structural variations along the chain. This is supported by the observation of domains placed at irregular distances from each other. Whereas the average particle size is below the single-domain critical diameter *D*_c_ ≈ 80 nm for Co-fcc, there are large multi-domain particles along the chain that could act as nucleation centers that initiate the domain formation as observed in diameter-modulated cylindrical NWs.^79^ Moreover, different directions of the MCA could be expected along the chain as the NPs have not fused to form a NW.

To gain insights into the mechanisms behind the domain formation, micromagnetic simulations were performed on NCs made up of NPs ranging from 30 to 50 nm, with and without the inclusion of a larger 100 nm multi-domain particle and also considering uniform and random MCA orientations. The magnetization configurations were obtained by minimizing the energy after saturating the NC perpendicular to the chain axis which is shown in Fig. 5(e) and (f). In addition, the equilibrium magnetization configuration for a NW, with fused particles, was simulated in the same way shown in Fig. 5(g). The simulations suggest that the randomly oriented MCA results in domain formation along the chains. Morover, when including a larger NP in the chain, the domain wall is preferably formed in the multi-domain particle in the form of a vortex. In contrast, domains are not formed in NCs or NWs when considering uniform MCA with longitudinal easy-axis (Fig. 5(f) and (g)), suggesting that the domain formation with irregular distances in Fig. 5(d) is governed by the randomness of the MCA, and local variations in the size of particles along a chain.

**Fig. 6:**
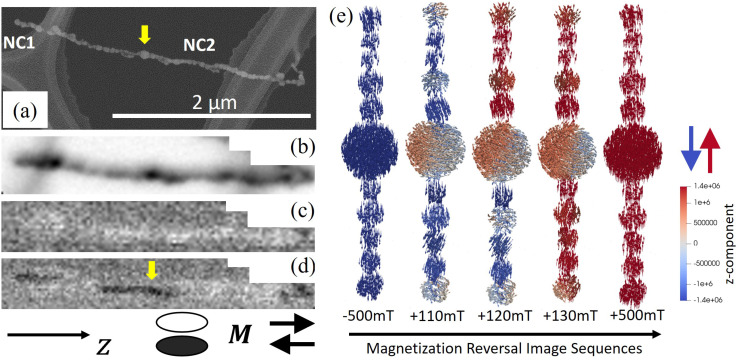
(a) SEM image of a short (NC1) and a long (NC2) chain. (b) STXM image from the same area. Magnetic contrast after magnetizing along (c) +*z* and (d) −*z* using *in situ* fields of ±140 mT. (e) Magnetization reversal sequences while sweeping the field from −500 to +500 mT along the chain axis. The red–blue color code represents the projection of the magnetization along the *z*-direction parallel to the chain axis. Note that the particle magnetization, given by the arrows, can also have *x*–*y* components during the reversal, which will be important for the magnetotransport properties of the NCs.

Furthermore, the magnetization reversal of individual NCs was studied using STXM and *in situ* magnetic fields applied along the chain-axis. Fig. 6(a) and (b) shows an SEM and STXM image from the same two NCs, one short composed of 4 NPs (NC1) and one much longer with a length of about 2 μm (NC2). The STXM image clearly reproduces the structural features with the larger NPs along the longer chain clearly visible. Fig. 6(c) displays the spatially resolved XMCD signal while applying an in-plane 140 mT *in situ* magnetic field along the positive *z*-direction. Note that the *in situ* field is parallel to the field applied during the self-assembly and thus also aligned with the remanent magnetization. As expected, the resulting magnetic contrast demonstrates a homogeneous magnetization along the NC parallel to the applied field and the remanent magnetization (bright contrast). Reversing the magnetic field results in the shorter chain (NC1) switching its magnetization (dark contrast), see Fig. 6(d). The switching can be explained by the small shape anisotropy of the shorter chain, allowing the weak *in situ* field of −140 mT to reverse the magnetization. Magnetization switching under the −140 mT field is also observed in a region along NC2 where switching occurs in the middle of the chain close to a significantly larger NP, indicated by arrows in Fig. 6(a) and (d).

To further study the impact of multi-domain particles during the magnetization reversal process in NCs, micromagnetic simulations were conducted on a chain with an inclusion of a 100 nm particle. Fig. 6(e) selectively illustrates the magnetization configuration of the NC as the magnetic field is swept from −500 mT to +500 mT along the chain (*z*-direction). The results reveal that the magnetization reversal starts from variations in the spin structure of the large particle shell and also particles located at the ends of the chain. The process continues with the formation of a vortex inside the larger particle, the emergence of transverse magnetization configurations in the NPs at 110 mT, and magnetization switching of the half of the NC at around 120 mT. By further increasing the field, the other half of the chain reverses its magnetization along the +*z*-direction, at +500 mT the system reaches saturation with the magnetization lying along the chain axis. The results are consistent with the STXM-XMCD measurements in Fig. 6 and emphasize the effect of multi-domain particles in the magnetization reversal.

#### 3.2.3. Direct integration and magnetotransport

**Fig. 7:**
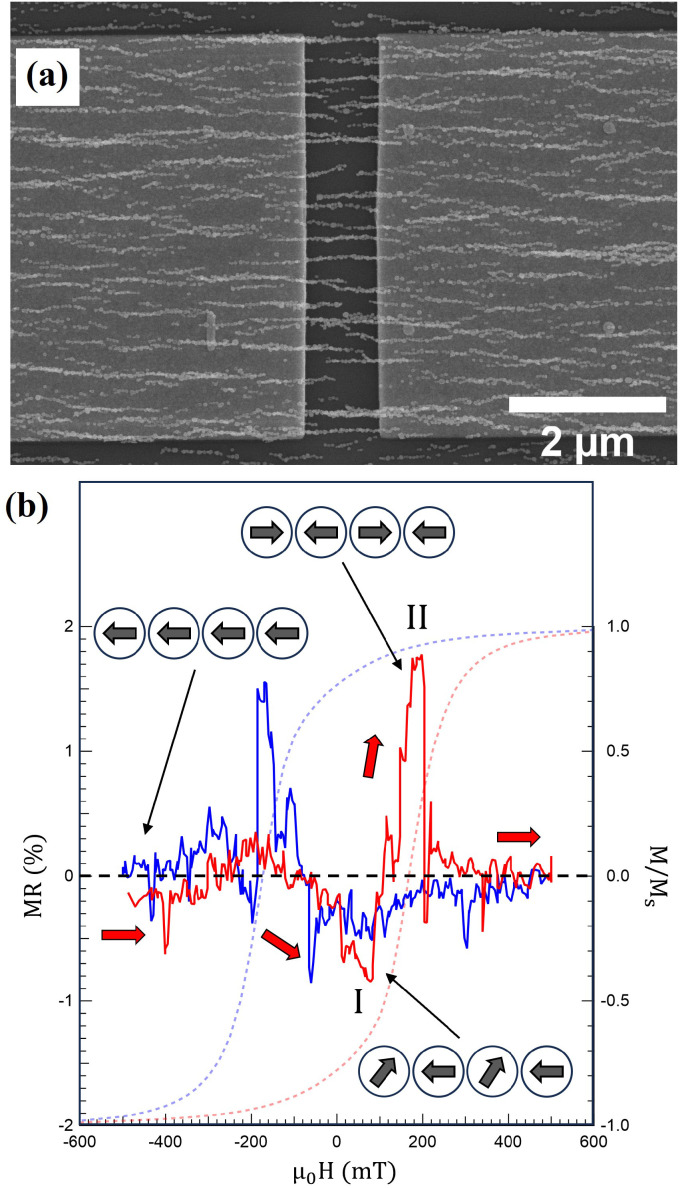
(a) SEM image of Co NCs directly self-assembled across a 1 μm gap between the source and drain. (b) MR behavior measured using a constant bias voltage of −0.5 V while varying a magnetic field along the chain axis at 25 mK. The arrows and schematic magnetization configurations correspond to sweeping the magnetic field from −500 mT in the positive direction. Similar behavior is observed when sweeping the field in the opposite direction (blue MR curve), but arrows and schematic magnetization configuration has been omitted for clarity. Also shown is a hysteresis loop recorded from S2 at 2 K.

Fig. 7(a) shows the direct self-assembly of Co nanoparticles across a 1 μm gap separating two Au pads acting as source and drain electrodes. It is observed that 9 NCs are bridging the contacts in this device. The system is analogous to particle film structures,^34,62–64^ but with a 1D arrangement of the NPs (nanograins). The magnetoresistance, MR = Δ*R*/*R*_sat_, of the device was measured while applying a magnetic field along the chain axis at a temperature of 25 mK, and the result is shown in Fig. 7(b), together with a hysteresis loop recorded from S2 at 2 K to correlate MR and magnetization changes. Starting from a saturated state *R*_sat_ at *μ*_0_*H* = −500 mT and sweeping the field in the positive direction results in a gradual decrease of the MR with a minimum denoted by I in Fig. 7(b). The behavior is ascribed to a reduced scattering cross-section due to the formation of transverse magnetization components as the NPs start reversing their magnetization along the NCs, similar to previous studies of polycrystalline Co NWs.^80^ The simulated reversal process at 110 and 120 mT in Fig. 6(e) shows examples of the emergence of perpendicular magnetization components in the NPs as the magnetization rotates away from the chain axis. The rotation continues until the formation of magnetic domains at the nucleation field *H*_n_ ≈ 100 mT, where a steep increase with a peak of MR = 1.8% centered around the coercive field *μ*_0_*H*_c_ ≈ 200 mT is observed (II in Fig. 7(b)). An example of magnetic domain formation during the magnetization reversal can be seen in the STXM images in Fig. 6(d). The increase of MR at *H*_c_ is attributed to the formation of magnetic domains along the NCs that, on average, have opposite magnetization, leading to an increased resistivity as the electrons travel across regions with antiparallel configuration, similar to granular particle systems.^34,62,64^ Furthermore, domain walls can further contribute to the increase in resistivity due to spin accumulation, as previously observed for Co NWs with similar diameters.^81^

The device in Fig. 7 is one out of seven produced on the same Si chip, of which six exhibit MR behavior with changes in the resistivity around the nucleation and coercive fields, see Fig. S4.† It should be noted that the source and drain are contacted through a set of parallel unique NCs with different nanoscale structures. As demonstrated, variations in the MCA and the size of the NPs along the NCs influence the domain formation and magnetization reversal. Subsequently, the two MR contributions denoted I and II above can vary in magnitude between the devices. The main contribution for devices 1 and 4 comes from domain formation at *H*_c_ with an increase in MR, whereas a decrease due to transverse magnetization components dominates in devices 2 and 5. For device 3, both mechanisms contribute equally, and for device 6, a more complex MR behavior is observed.

## 4. Conclusions

This work presents the first magnetotransport study of metallic NCs integrated into devices. The magnetoresistive elements were directly integrated using a novel template-free approach with directed self-assembly of gas-phase generated NPs using a combination of electric and magnetic fields. Measuring the electric conductance reveals an MR of 0.5–1.8%, providing proof of principle for facile fabrication of magnetoresistive devices based on 1D nanogranular sensing elements. Furthermore, the study presents the first investigation into the effect on the magnetic properties as 1D nano-granular systems are transformed into single-crystalline NWs. By combining magnetometry, X-ray microscopy, and micromagnetic simulations, we demonstrate how interparticle interactions, local variations in the MCA, and the size of the individual NPs play a crucial role in domain formation and magnetization reversal.

## Author contributions

Mehran Sedrpooshan: Conceptualization, investigation, data curation, formal analysis, writing – original draft. Claudiu Bulbucan: Investigation, data curation, formal analysis, writing – review & editing. Pau Ternero: Investigation, data curation, writing – review & editing. Pierfrancesco Maltoni: Investigation, data curation, formal analysis, writing – review & editing. Calle Preger: Investigation, formal analysis, writing – review & editing. Simone Finizio: Data curation, writing – review & editing. Benjamin Watts: Data curation, writing – review & editing. Davide Peddis: Writing – review & editing, supervision, funding acquisition. Adam M. Burke: Investigation, formal analysis, writing – review & editing, supervision. Maria E. Messing: Conceptualization, writing – review & editing, supervision, funding acquisition. Rasmus Westerström: Conceptualization, formal analysis, writing – original draft, supervision, funding acquisition.

## Conflicts of interest

The authors declare no potential conflict of interest.

## Supplementary Material

NR-015-D3NR03878E-s001
