# Supplementary material for: Template-free generation and integration of functional 1D magnetic nanostructures
Source: Nanoscale. 2023 Nov 9;15(45):18500–10. doi: 10.1039/d3nr03878e (PMC10667589; doi:10.1039/d3nr03878e)
Supplement: NR-015-D3NR03878E-s001 [file NR-015-D3NR03878E-s001.pdf]

Electronic Supplementary Material (ESI) for Nanoscale.
This journal is © The Royal Society of Chemistry 2023

Supplementary Information:

Template-Free Generation and Integration of Functional 1D Magnetic Nanostructures

Mehran Sedrpooshan,[a,b] Claudiu Bulbucan,[c] Pau Ternero,[a,d] Pierfrancesco Maltoni,[e] Calle Preger,[c,f] Simone Finizio,[g] Benjamin Watts,[g] Davide Peddis,[h,i] Adam M Burke,[a,d] Maria E Messing,[a,b,d] Rasmus Westerström[a,b]

[a]NanoLund, Lund University, Box 118, 221 00 Lund, Sweden
[b]Synchrotron Radiation Research, Lund University, Box 118, 221 00 Lund, Sweden
[c]MAX IV Laboratory, Lund University, Lund, SE-22100, Sweden
[d] Solid State Physics, Lund University, Box 118, 221 00 Lund, Sweden
[e]Department of Materials Science and Engineering, Uppsala University, Box 35, 751 03 Uppsala, Sweden
[f] Ergonomics and Aerosol Technology, Lund University, Lund, SE-22100, Sweden
[g]Paul Scherrer Institut, 5232 Villigen PSI, Switzerland
[h]Institute of Structure of Matter, National Research Council (CNR), Monterotondo Scalo, 00015 Rome, Italy
[i]Department of Chemistry and Industrial Chemistry, University of Genova, 16146 Genova, Italy

(a)

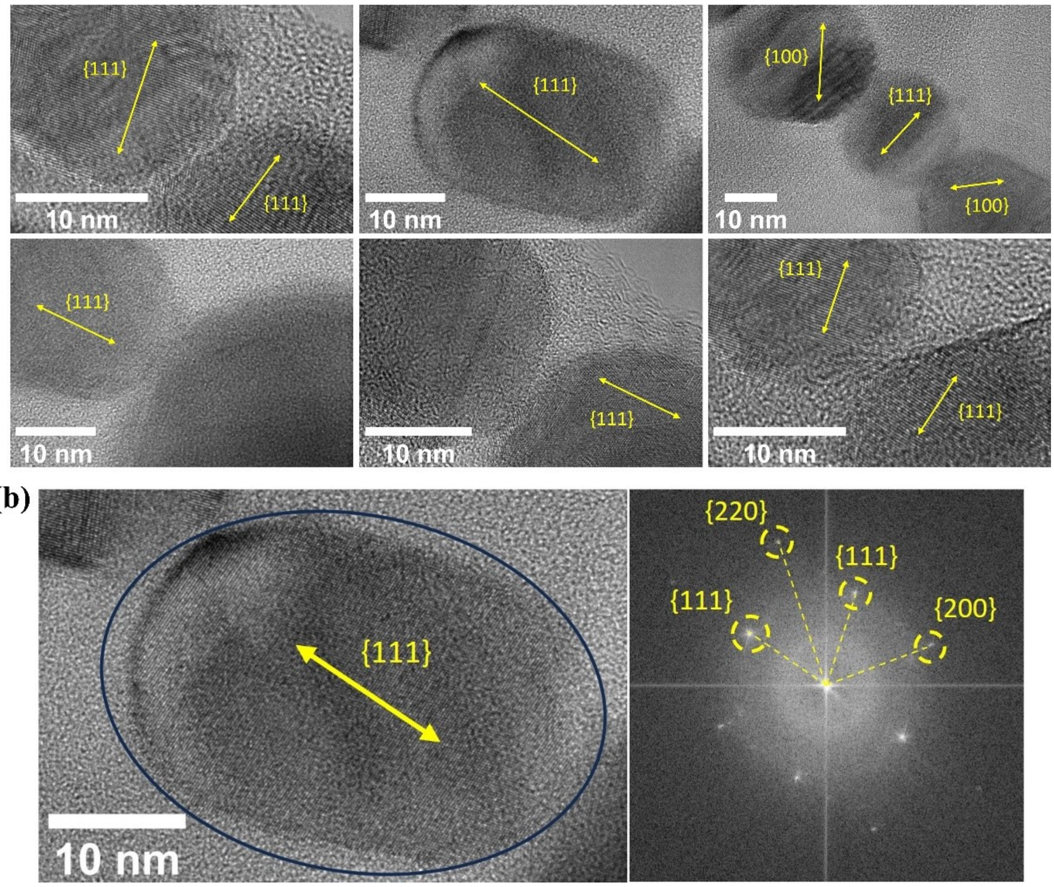

Fig. S1 a) HRTEM images showing single-crystalline Co fcc nanoparticles, b) HRTEM and corresponding fast Fourier transform diffractogram of a nanoparticle displaying a single crystalline fcc pattern.

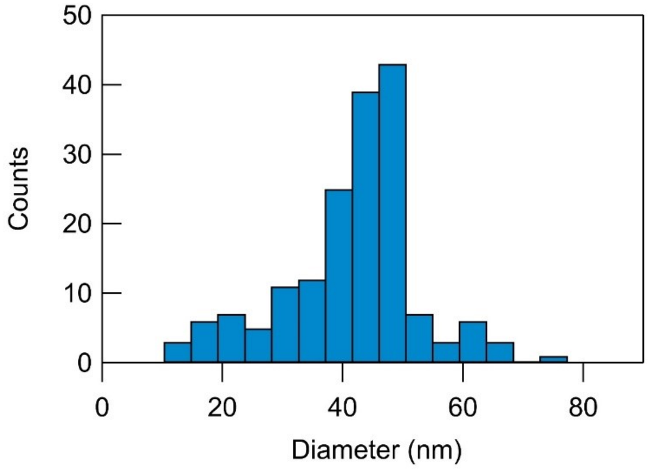

Fig. S2 Size distribution of Co nanoparticles. The histogram was obtained by SEM imaging and analysis of several regions of a low coverage sample. Based on the histogram, the produced particles have an average diameter of 41 nm which is consistent with the size of 40 nm set with the differential mobility analyzer.

.

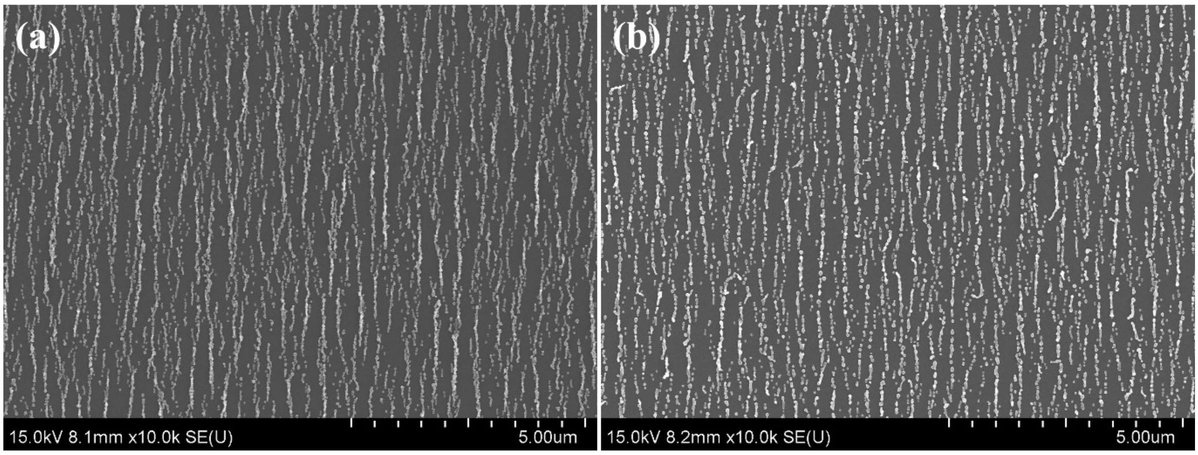

Fig. S3 Low-magnification SEM images of a) nanochains, and b) nanowires, used for obtaining histograms presented in Fig. 4. The analysis is done by thresholding and binarizing the images, calculating the structures' area and converting areas to lengths.

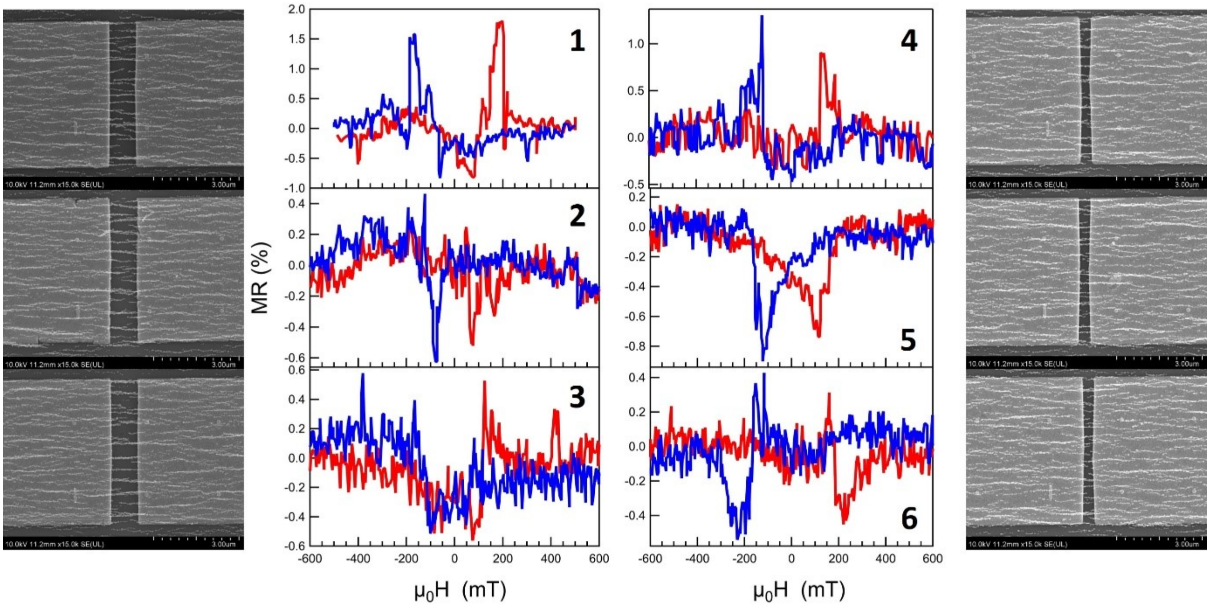

Fig. S4 MR behavior and SEM images of six devices. Devices 1-3 and 4-6 are fabricated with different gap distances of 1 µm and 500 nm, respectively.
